# Piezoelectric osteotomy in hand surgery: first experiences with a new technique

**DOI:** 10.1186/1471-2474-7-36

**Published:** 2006-04-12

**Authors:** Dominik J Hoigne, Stefan Stübinger, Oliver Von Kaenel, Sonia Shamdasani, Paula Hasenboehler

**Affiliations:** 1 Clinic for General Surgery, University Hospital of Basel, 4031 Basel, Switzerland; 2 Clinic for Reconstructive Surgery, University Hospital of Basel, 4031 Basel, Switzerland; 3 Clinic for Plastic-, Reconstructive-, and Handsurgery, Kantonsspital Aarau, 5001 Aarau, Switzerland; 4 Bethesda Hospital, 4020 Basel, Switzerland

## Abstract

**Background:**

In hand and spinal surgery nerve lesions are feared complications with the use of standard oscillating saws. Oral surgeons have started using a newly developed ultrasound bone scalpel when performing precise osteotomies. By using a frequency of 25–29 kHz only mineralized tissue is cut, sparing the soft tissue. This reduces the risk of nerve lesions. As there is a lack of experience with this technique in the field of orthopaedic bone surgery, we performed the first ultrasound osteotomy in hand surgery.

**Method:**

While performing a correctional osteotomy of the 5th metacarpal bone we used the Piezosurgery^® ^Device from Mectron [Italy] instead of the usual oscillating saw. We will report on our experience with one case, with a follow up time of one year.

**Results:**

The cut was highly precise and there were no vibrations of the bone. The time needed for the operation was slightly longer than the time needed while using the usual saw. Bone healing was good and at no point were there any neurovascular disturbances.

**Conclusion:**

The Piezosurgery^® ^Device is useful for small long bone osteotomies. Using the fine tip enables curved cutting and provides an opportunity for new osteotomy techniques. As the device selectively cuts bone we feel that this device has great potential in the field of hand- and spinal surgery.

## Background

For osteotomies of the hand oscillating saws are usually used [1]. Even though they are varied in size, they are not very precise for use in the vicinity of nerves and arteries. They also pose problems while being used in conjunction with magnification, as one's range of sight and focus is restricted when wearing magnifying glasses. For that reason oral surgeons have moved to using the newly developed piezoelectrical bone scalpel when operating in the near vicinity of nerves or arteries. The tip of this instrument oscillates in the frequency of ultrasound [2]. The mechanism of this device is based on the so called Piezo – Effect. French Physicists Jean and Marie Curie first mentioned the direct Piezo-Effect 1880, whereby certain crystals produce electrical current while under mechanical pressure. The reciprocal effect, by which the crystals are deformed when under electrical current, was then discovered a while later. This is the effect being used by the Piezosurgery Device^®^. In this device, the electrical field is located in the handle of the saw [3]. Due to the deformation caused by the electrical current, a cutting – hammering movement is produced at the tip of the instrument. These micro movements are in the frequency range of 25 to 29 kHz and, depending on the insert, with an amplitude of 60 to 210 μm. This way only mineralized tissue is selectively cut. Neurovascular tissue and other soft tissue would only be cut by a frequency of above 50 kHz [3-5]. Depending on the strength of the bone and the blade geometry, the efficiency of the cutting can be regulated by the frequency modulator and the power level. For cooling there is an integrated pump with five different working levels. This pump automatically washes physiological solution to the area being cut. The cost of the device is about 7.000 USD. Additional costs per operation are for the cooling liquid and are in the range of a few dollars. We have used the Piezosurgery Device^® ^by Mectron [Italy] [3] for the first time in osteotomies of the long bone in the field of hand surgery. We will report on our experience with one case, with a follow up time of one year.

## Method

**Figure 1:**
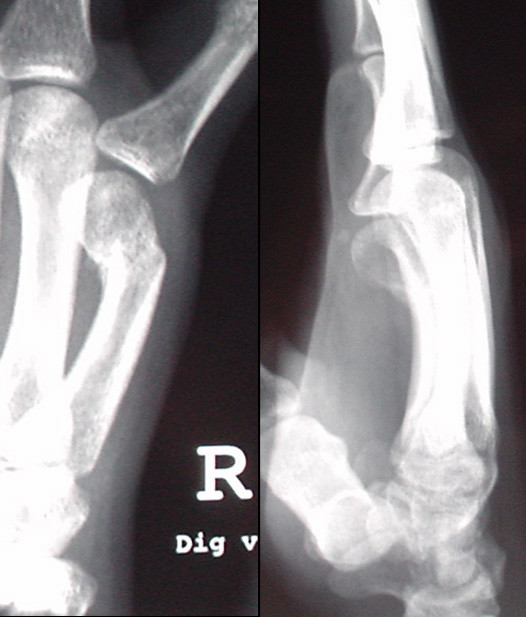
Malunion of the fifth metacarpal bone of the right hand.

**Figure 2:**
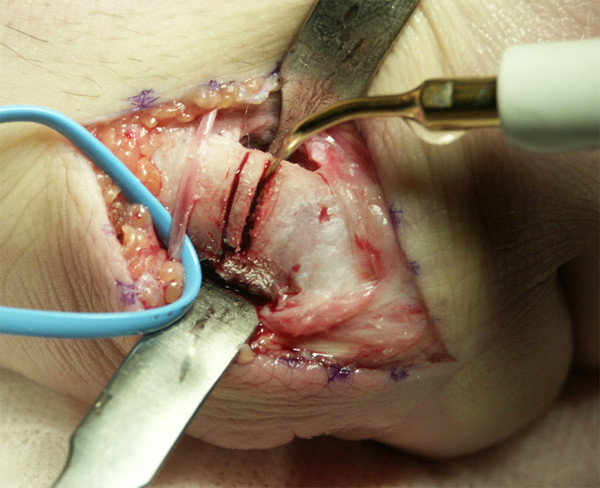
Closing wedge osteotomy using the Piezosurgery Device^®^.

**Figure 3:**
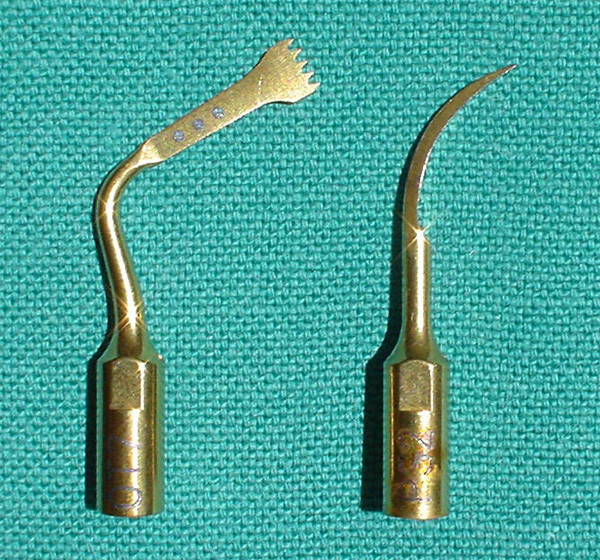
Two examples of available tips. Left side: The preferred blade.

The correctional osteotomy was performed on a 23 year old worker who suffered a malunited metacarpal bone fracture of the fifth finger on his dominant hand. The X-ray revealed a 45 degree angular deformity of the fifth metacarpal neck with internal rotation. (Figure 1). The operation was performed under regional anesthesia. A longitudinal incision was made over the fifth metacarpal. The tendon of the extensor digiti minimi was found and on its radial side the periosteum of metacarpal five was reached. The periosteum was opened longitudinally over the defect as usual. For the correction of the defect of 45 degrees, a bone wedge was excised. Instead of using the traditional oscillating saw, the Piezosurgery Device^® ^[3] was used (Figure 2). We used a sharp hardened saw coated with titannitrid (Figure 3). For most of the surgery the highest power level, the boosted burst c, was used. We set the automatic cooling of the area with water to its highest level. The angulation and rotation was corrected and fixed with a 1.5 mm titanium five-hole plate and four screws. Closure of the wound was done in layers. Mobilization was started on the 10^th ^postoperative day. The overall time of observation was one year.

## Results and discussion

**Figure 4:**
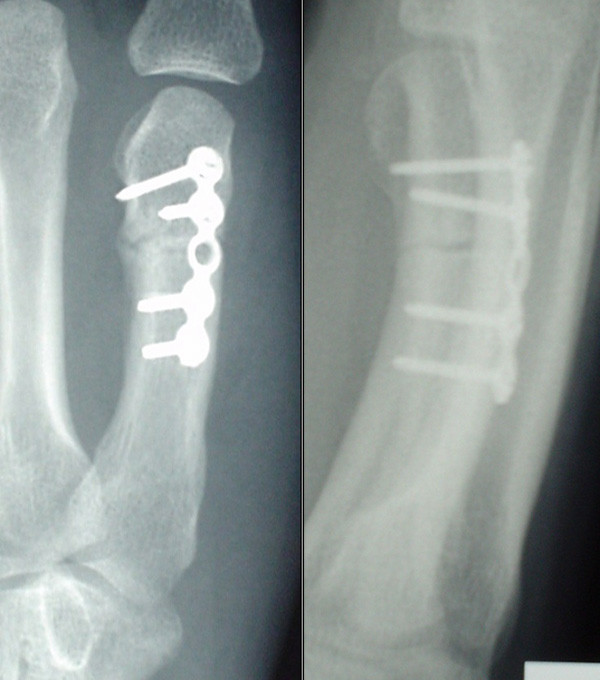
The correction obtained with closing wedge osteotomy.

The Piezosurgery^® ^Device is ideally sized for hand surgery. The cutting was very precise. The edges of the osteotomy were all sharp to the edge, there was no need to split the bone with a chisel, nor was there the danger of a break out. During the osteotomy there were no disturbing vibrations in the area of operation. This absence of vibration is very practical for operations using a magnifier. Vercellotti mentions that to overcome any problems during surgery, instead of increasing pressure on the hand piece, as in traditional techniques, it is necessary to find the correct pressure to achieve the desired result. With piezoelectric surgery, increasing the working pressure above a certain limit impedes the vibrations of the insert [4]. We have also experienced this in our study. The instrument can be moved in all directions comparable to a pen. The tip of the instrument is exchangeable. Using the fine tip enables multiplanar as well as curved cutting. Because of the automatic water cooling during the whole procedure, there is always a clear view onto the object. This is something oral surgeons found especially useful [6]. The authors mention that the downside of the device is the relative slow sawing process. We needed about 30 seconds for one cut of the relatively small bone. This is about 20 seconds longer than the time needed for cutting with the usual saw. Although the power can be regulated with the power box and the use of different scalpels, we agree with other authors that the optimal use of this device is in surgeries of small bones where precise and soft tissue friendly cutting is required [7]. As other literature has shown, the device selectively cuts bone while sparing nerves and other soft tissue [2,3]. This allows for minimal invasive surgeries with limited retraction of soft tissue and minimal stripping of the periosteum, saves time and might have a positive effect on the healing process. Our aim of the first time use of the Piezosurgery^® ^Device in hand surgery was to check its usability in osteotomies of tubular bones. The preparation of the bone was done in the usual manner as is done when cutting with an oscillating saw. The reason for this was to fully visualize the cutting process using this new device, although in the future, it should be possible to minimize the bony exposure. In our patient the postoperative healing of the wound and the bone consolidation (Figure 4) were smooth. The duration of postoperative sick leave was four weeks which is more rapid than the usual recovery period. The patient regained full use of his finger according to the state before the fracture. At no point was there any loss of sensitivity. The patient as well as the surgeons were fully satisfied with the result.

## Conclusion

The Piezosurgery^® ^Device is a useful device for small long bone osteotomies. We feel that this device has great potential in the field of hand- and spinal surgery. As the device selectively cuts bone, considerable nerve lesions can be avoided and minimal invasive surgeries are possible. Using the fine tip enables curved cutting and provides an opportunity for new osteotomy techniques.

## Competing interests

The author(s) declare that they have no competing interests.

## Authors' contributions

DJH initiated and coordinated the new application of Piezosurgery^® ^device and wrote the publication.

StSt lead the osteotomy as he was experienced with this tool from oral surgery. He played a major part in writing the technical aspects.

OVK was the treating surgeon, performed the operation and evaluated the new tool.

SS performed a literature review and wrote part of the publication.

PH was the treating chief surgeon, evaluated the new tool and lead the treatment in all aspects.

## Consent

We obtained oral consent from the patient but could not obtain written consent.

## Pre-publication history

The pre-publication history for this paper can be accessed here:
